# Prosthetic Valve Function after Aortic Valve Replacement for Severe Aortic Stenosis by Transcatheter Procedure versus Surgery

**DOI:** 10.3390/jcdd9100355

**Published:** 2022-10-16

**Authors:** Shunsuke Saito, Toshimi Sairenchi, Shotaro Hirota, Ken Niitsuma, Shohei Yokoyama, Yasuyuki Kanno, Yuta Kanazawa, Masahiro Tezuka, Yusuke Takei, Go Tsuchiya, Taisuke Konishi, Ikuko Shibasaki, Koji Ogata, Osamu Monta, Yasushi Tsutsumi, Hirotsugu Fukuda

**Affiliations:** 1 Department of Cardiac and Vascular Surgery, Dokkyo Medical University, 880 Kitakobayashi, Mibu 321-0293, Japan; 2 Medical Science of Nursing, School of Nursing, Dokkyo Medical University, 880 Kitakobayashi, Mibu 321-0293, Japan; 3 Department of Cardiovascular Surgery, Fukui Cardiovascular Center, Fukui 910-0833, Japan

**Keywords:** aortic stenosis, transcatheter aortic valve replacement, surgical aortic valve replacement, patient–prosthesis mismatch, paravalvular leakage

## Abstract

**Background** This study compared the clinical outcomes of transcatheter (TAVR) and surgical (SAVR) aortic valve replacements, focusing on postoperative valvular performance assessed by echocardiography. **Method and Results** A total of 425 patients who underwent TAVR (230 patients) or SAVR (195 patients) were included. Postoperative effective orifice area index (EOAI) was higher in the TAVR group (1.27 ± 0.35 cm^2^/m^2^) than in the SAVR group (1.06 ± 0.27 cm^2^/m^2^, *p* < 0.001), and patient–prosthesis mismatch (PPM) was more frequent in the SAVR group (22.6%) than in the TAVR group (8.7%, *p* < 0.001). Mild or greater paravalvular leakage (PVL) was more frequent in the TAVR group (21.3%) than in the SAVR group (0%, *p* < 0.001). Moreover, there was no difference in freedom from all-cause death, stroke, or rehospitalization between the groups. Patients with moderate or greater PPM (EOAI < 0.85 cm^2^/m^2^) had lower freedom from composite events than those without this PPM criterion (*p* = 0.008). Patients with mild or greater PVL also had lower freedom from composite events than those without this PVL criterion (*p* = 0.017). **Conclusions** Postoperative valvular performance of TAVR was superior to that of SAVR in terms of EOAI. This merit was counterbalanced by the significantly lower rates of PVL in patients who underwent SAVR. The overall clinical outcomes were similar between the study groups.

## 1. Introduction

Aortic stenosis (AS) is the most common heart valve disease in developed countries. Surgical aortic valve replacement (SAVR) has long been the gold standard for the surgical treatment of aortic valve disease, with well-documented benefits in terms of symptom improvement and survival [1,2]. Over the last decade, transcatheter aortic valve replacement (TAVR) has revolutionized the treatment of severe AS. Early studies showed a clear benefit of TAVR in prohibitive and high-surgical-risk patients (Society of Thoracic Surgery-Predicted Risk of Mortality [STS-PROM] > 8%) [3,4,5], and intermediate-risk patients (STS-PROM 4%–8%) [6,7]. In addition, two comparative trials on low-risk patients reported promising results [8,9]. Most guidelines have derived their current indications from these industry-driven randomized controlled trials (RCTs) [10,11].

Both valvular replacement techniques could be associated with operative functional complications, such as patient–prosthesis mismatch (PPM) and paravalvular leakage (PVL). PPM occurs when high transvalvular gradients remains postoperatively due to an imbalance between the aortic prosthetic size and the orifice area required for an adequate blood perfusion [12]. PPM is associated with diminished regression of the left ventricular mass, increased incidence of bioprosthetic valve dysfunction, symptom recurrence, and unfavorable clinical outcomes [13]. Additionally, mortality and the incidence of early structural valve deterioration increase in patients with PPM after aortic valve replacement (AVR) compared with those in patients without PPM [14,15]. Depending on its degree, PVL after AVR leads to left ventricular volume overload, which indirectly affects pulmonary circulation and is associated with increased postoperative morbidity and mortality [16].

Although TAVR has revolutionized the surgical treatment for severe AS, with a dramatic increase in the number of patients who underwent this treatment in the last decade, the precise incidence of PPM and PVL as well as their impact on the long-term clinical outcomes have not been fully understood. Therefore, this study aimed to compare the clinical outcomes of TAVR and SAVR, focusing on postoperative valvular performance assessed by echocardiography.

## 2. Methods

### 2.1. Patients

Ethics approval was obtained from the appropriate ethics committee for this retrospective study. The requirement for individual consent was waived. From October 2015 to July 2019, 678 patients underwent AVR at our institute. Of the 678 patients, 224 who underwent AVR concomitantly with aortic or mitral surgery, left ventricular restoration, left ventricular assist device implantation, or AVR for aortic insufficiency or infective endocarditis were excluded from this study. In the postoperative echocardiographic study, data regarding the effective orifice area (EOA) were missing for 29 patients, who were also excluded. Finally, the remaining 425 patients, who underwent AVR for severe AS, with adequate postoperative echocardiographic data were included in this study.

### 2.2. Treatment Selection

The selection of treatment (TAVR or SAVR) is performed through discussions within the heart team including cardiologists, surgeons, anesthetists, nurses, and clinical engineers. Transcatheter or open surgical approach was selected in consideration of patient age, condition severity, frailty, cognitive function, and comorbidities. All procedures were performed under general anesthesia and using transesophageal echocardiography. The approach in the TAVR group (transfemoral, transapical, or other alternative approaches) was selected through discussions within the heart team in consideration of the anatomical characteristics of the patient. SAVR was performed through median sternotomy and under cardiac arrest with cardiopulmonary bypass support.

### 2.3. Data Collection

Data were extracted from patient charts recorded in the hospital’s computer database. Follow-up started on the day of TAVR or SAVR. Postoperative echocardiographic study was conducted 1 week after procedure in most patients. In some patients in TAVR group, echocardiography was also conducted 1 day after procedure to rule out complications, such as cardiac tamponade. The initial postoperative echocardiographic data were used for the analysis.

### 2.4. Endpoints

The primary endpoint was a composite of all-cause death, stroke, or rehospitalization. Patients with suspected stroke after procedure underwent computed tomography and/or magnetic resonance imaging study. Those with newly detected lesions in these studies were diagnosed with stroke. Rehospitalization was defined as any hospitalizations related to the procedure, the valve, or heart failure. The key secondary endpoints were all-cause death, stroke, and rehospitalization.

### 2.5. Statistical Analysis

Continuous variables are presented as mean ± SD and categorical variables as numbers and proportions. All continuous variables were examined for normal distribution using the Shapiro–Wilk test and a normal probability plot. For univariate analyses, normally distributed variables were compared using Student’s *t*-test, non-normally distributed variables were compared using the Mann–Whitney U-test, and categorical variables were compared using chi-square analysis or Fisher’s exact test, as appropriate. Differences in echocardiographic data (peak velocity, mean and peak pressure gradient, and EOA index [EOAI]) among valve sizes were analyzed using Spearman’s rank correlation coefficient. Time-to-event analyses were performed using Kaplan–Meier estimates and compared using the log-rank test. The multiplicity in pairwise comparisons was corrected using the Holm procedure. Risk factors for the primary endpoint were evaluated using a Cox hazard model. Factors with a *p*-value of <0.10 in the univariate analysis were included in the multivariable analysis. All two-sided *p*-values of <0.05 were considered statistically significant.

All statistical analyses were performed using EZR (Saitama Medical Center, Jichi Medical University, Saitama, Japan), a graphical user interface for R (The R Foundation for Statistical Computing, Vienna, Austria). More precisely, it is a modified version of R commander designed to add statistical functions frequently used in biostatistics [17].

## 3. Results

### 3.1. Patient Demographics

Of the total 425 patients, 230 underwent TAVR and 195 underwent SAVR. The preoperative characteristics of the TAVR and SAVR groups are summarized in Table 1. Mean age was significantly higher in the TAVR group (84.6 ± 4.3 years) than in the SAVR group (74.0 ± 8.0 years, *p* < 0.001). Compared with the SAVR group, there were more female patients in the TAVR group (72.2% vs. 42.1%, *p* < 0.001), while the body mass index was lower in the TAVR group (22.2 ± 3.7 vs. 23.4 ± 3.9 kg/m^2^, *p* < 0.001). Preoperative STS-PROM was higher in the TAVR group than in the SAVR group (6.6 ± 4.6 vs. 5.1 ± 5.5, *p* < 0.001). Furthermore, coronary artery diseases were more prevalent in the SAVR group (*p* < 0.001). In contrast, the TAVR group had a higher frequency of cerebral vascular disease or carotid artery disease (*p* = 0.018). TAVR was prohibited for hemodialysis patients in Japan during the study period; therefore, the proportion of patients with renal dysfunction and on hemodialysis was higher in the SAVR group (*p* < 0.001). There were more patients with bicuspid aortic valve in the SAVR group than in the TAVR group (20.0% vs. 2.6%, *p* < 0.001). The preoperative mean left ventricular ejection fraction was higher in the TAVR group (58.1% ± 11.4%) than in the SAVR group (55.7% ± 13.1%, *p* = 0.043). Emergency and urgent operations were more frequent in the SAVR group (*p* < 0.001), with concomitant or serial coronary revascularization being performed more frequently in the SAVR group (*p* < 0.001). The prostheses used in the TAVR and SAVR groups are summarized in Table 2.

### 3.2. Operative Outcomes

The operative outcomes of the TAVR and SAVR groups are summarized in Table 3. Postoperative intra-aortic balloon pumping was required more frequently in the SAVR group (7.2%) than in the TAVR group (1.3%, *p* = 0.002). The SAVR group also had more cases of intraoperative bleeding (*p* < 0.001), requiring more transfusions (*p* < 0.001). New-onset atrial fibrillation was observed in 30.3% patients in the SAVR group and only 5.7% in the TAVR group (*p* < 0.001). The majority of TAVR patients (79.6%) were extubated in the operating room, while none of the SAVR patients were extubated (*p* < 0.001). The duration of intensive care unit stay was significantly longer in the SAVR group than in the TAVR group (3.2 ± 3.8 vs. 1.4 ± 3.5 days, *p* < 0.001). Moreover, the TAVR group (10.0%) had a higher proportion of patients requiring postoperative permanent pacemaker implantation than the SAVR group (1.5%, *p* < 0.001). Peripheral vascular complications were only observed in the TAVR group (6.5% vs. 0%, *p* < 0.001).

### 3.3. Postoperative Echocardiographic Findings

The postoperative echocardiographic findings in the TAVR and SAVR groups are summarized in Table 3. Peak velocity through prosthesis was significantly higher in the SAVR group (2.3 ± 0.5 m/s) than in the TAVR group (2.1 ± 0.5 m/s, *p* < 0.001). Similarly, the mean and peak pressure gradients were significantly higher in the SAVR group (11.6 ± 4.9 and 21.4 ± 8.7 mmHg, respectively) than in the TAVR group (10.0 ± 4.8 and 18.5 ± 8.5 mmHg, respectively, *p* < 0.001 for both). Meanwhile, EOAI was higher in the TAVR group (1.27 ± 0.35 cm^2^/m^2^) than in the SAVR group (1.06 ± 0.27 cm^2^/m^2^, *p* < 0.001). PPM occurred more frequently in the SAVR group than in the TAVR group. Moderate PPM (EOAI < 0.85 cm^2^/m^2^) was observed in 22.6% of the SAVR group compared with 8.7% of the TAVR group (*p* < 0.001). Severe PPM (EOAI < 0.65 cm^2^/m^2^) also tended to be seen more frequently in the SAVR group (3.6% vs. 0.9%, *p* = 0.087). In contrast, PVL was significantly more frequent in the TAVR group (87.4% vs. 8.1%, *p* < 0.001). Similarly, mild or greater PVL was observed more frequently in the TAVR group (21.3%) than in the SAVR group (0%, *p* < 0.001).

**Figure 1:**
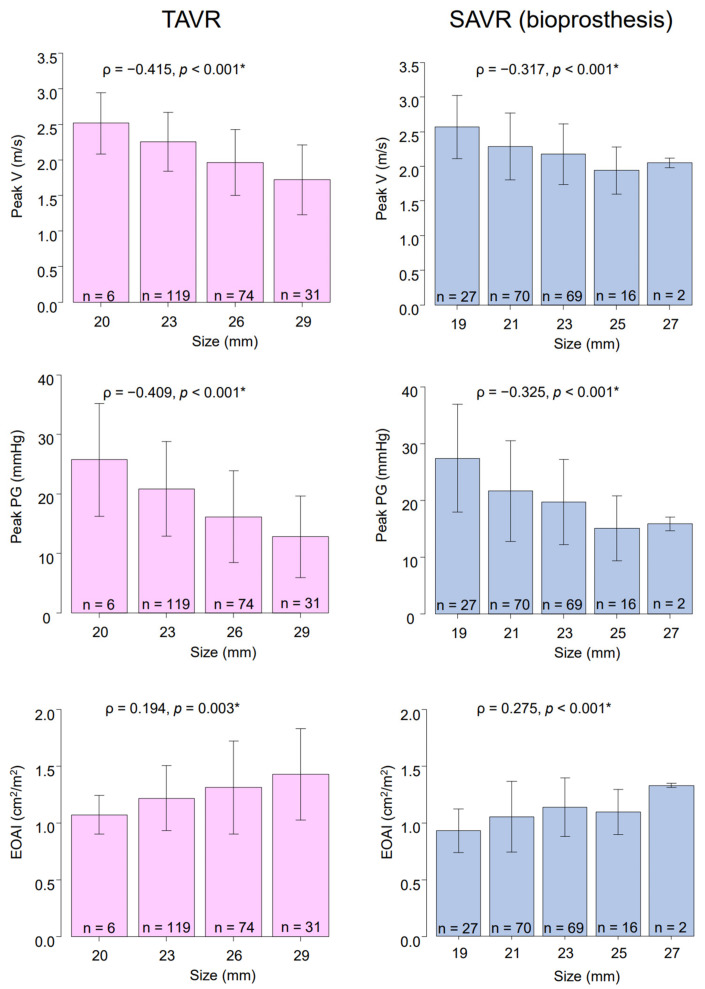
Prosthetic valvular performances stratified by the valvular sizes. EOAI: effective orifice area index, PG: pressure gradient, SAVR: surgical aortic valve replacement, TAVR: transcatheter aortic valve replacement, V: velocity. * Spearman’s rank correlation coefficient.

The prosthetic valvular performances, stratified by valvular sizes, in both groups are summarized in Figure 1. In general, a larger valvular size was associated with a lower peak velocity and peak pressure gradient. Furthermore, a larger valvular size is also associated with a higher EOAI. The mean EOAI of the 19 mm surgical bioprosthesis was 0.92 ± 0.19 cm^2^/m^2^.

**Figure 2:**
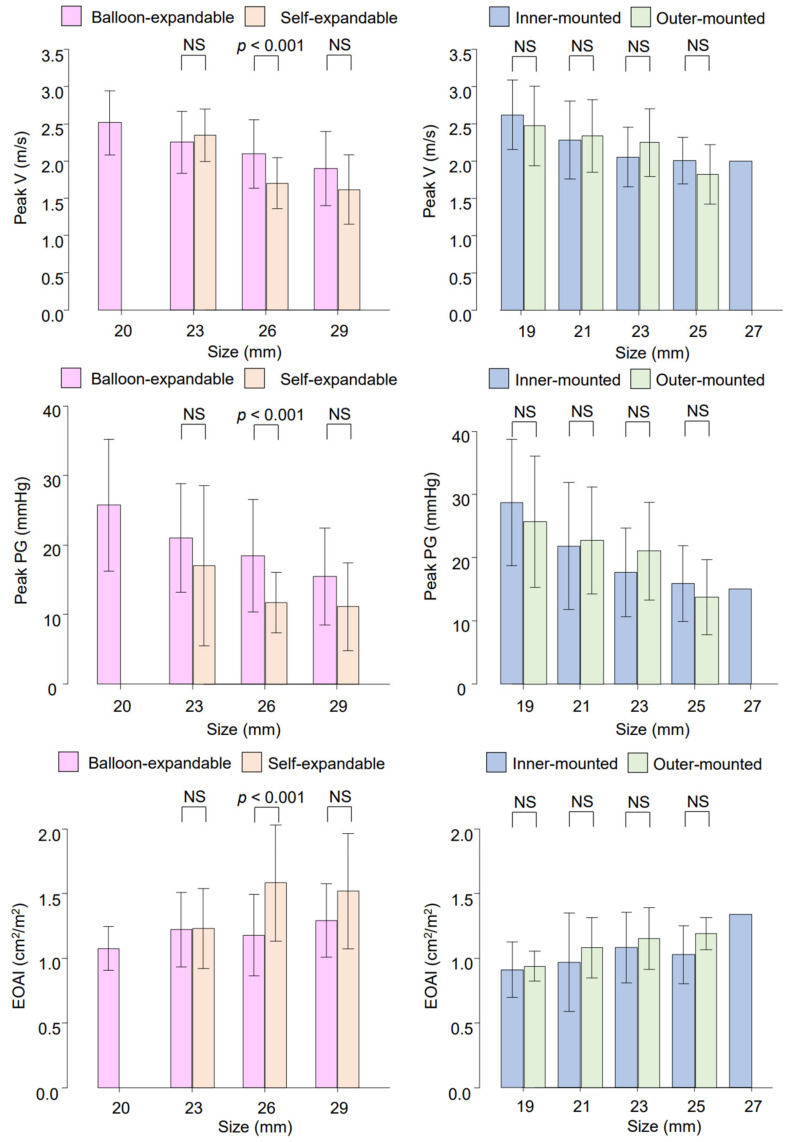
Valvular performances among different prosthetic types. EOAI: effective orifice area index, NS: not significant, PG: pressure gradient, V: velocity.

Valvular performance was further compared between the different prosthetic types (Figure 2). Among the transcatheter prostheses, self-expandable valves were compared with balloon-expandable valves in each size (i.e., 23, 26, 29 mm self-expandable valves vs. 23, 26, 29 mm balloon-expandable valves). Statistical significance was found only in the 26 mm valves, with a significant association of the self-expandable valves with lower peak velocity, lower peak pressure gradient, and higher EOAI (*p* < 0.001 for three parameters) (Figure 2). Furthermore, the most frequently used inner-mounted pericardial valves (Magna Ease, INSPIRIS RESILIA, and Carpentier-Edwards PERIMOUNT) were compared with the most frequently used outer-mounted pericardial valves (Crown PRT and Mitroflow). There was no significant difference between the inner- and outer-mounted valves of any sizes in any of the parameters (Figure 2).

### 3.4. Primary Endpoint

**Figure 3:**
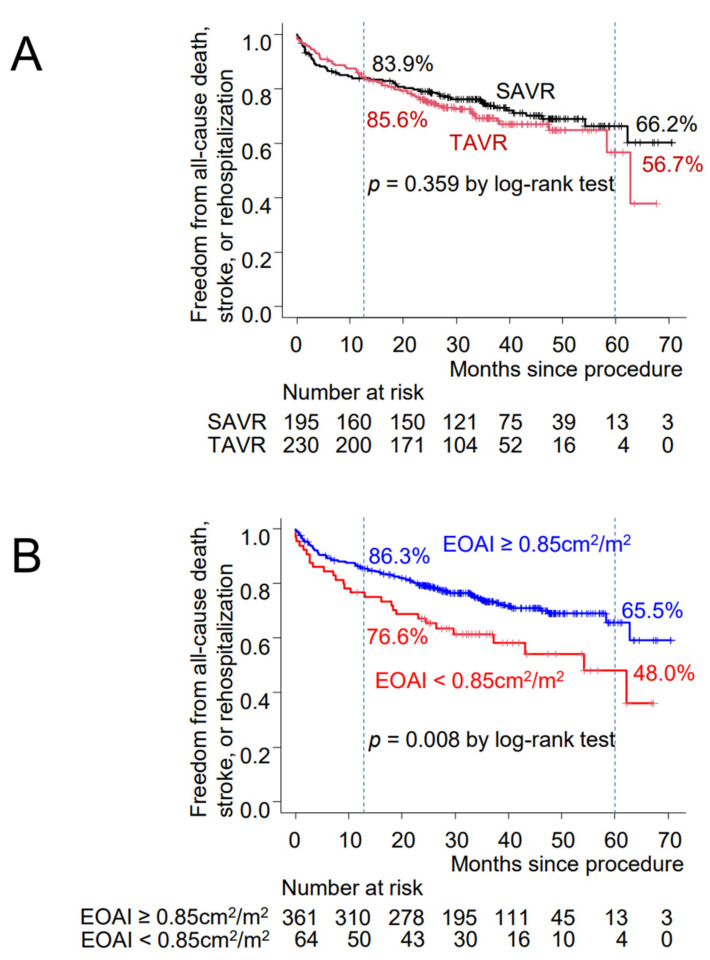

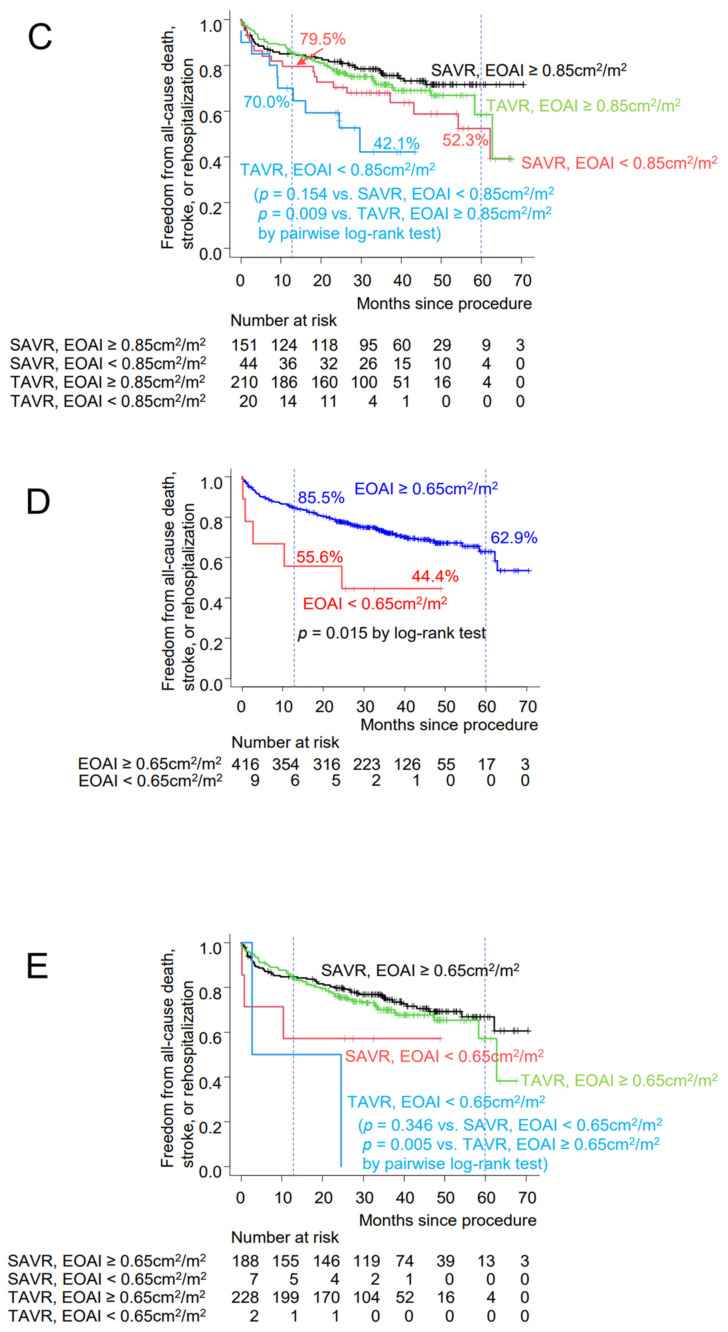

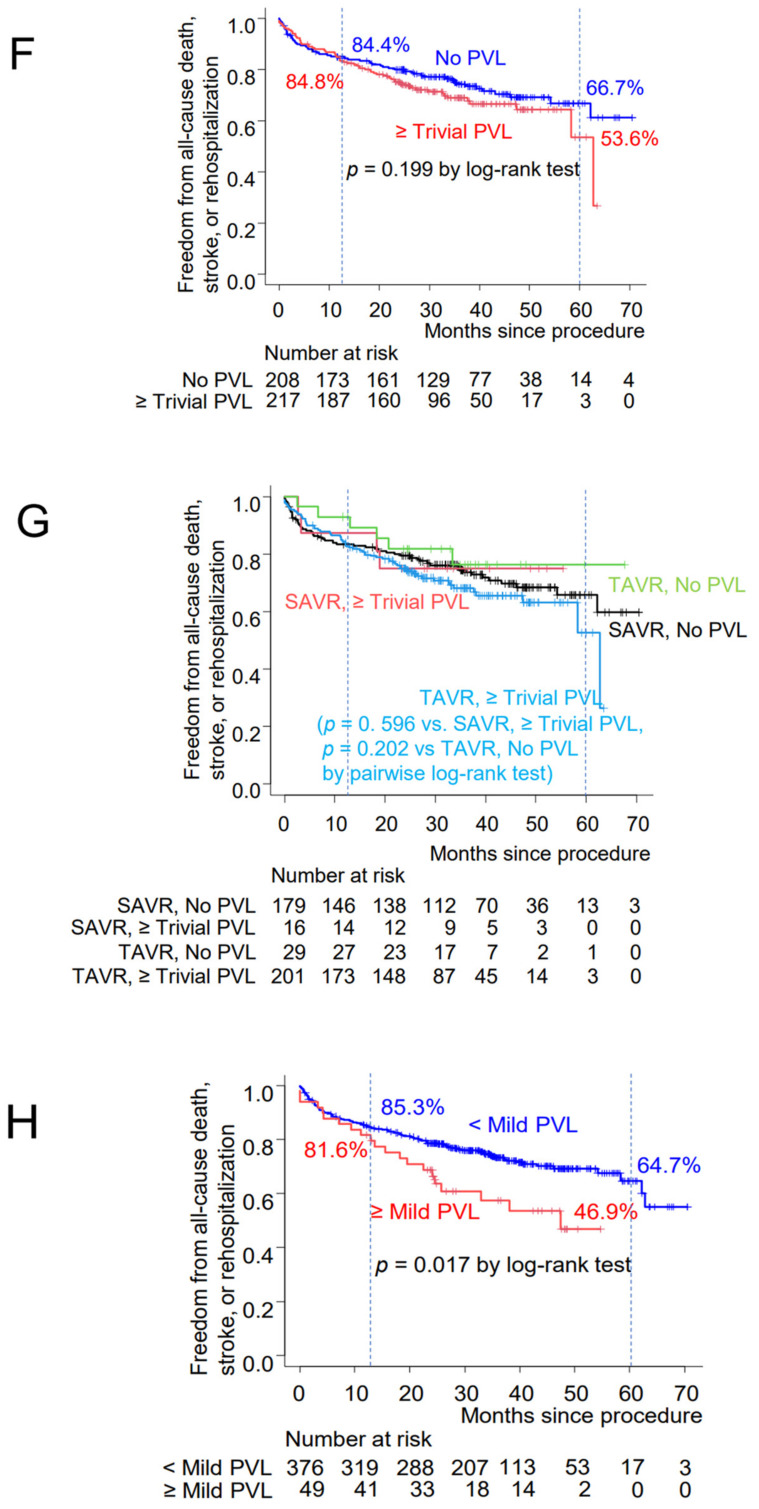

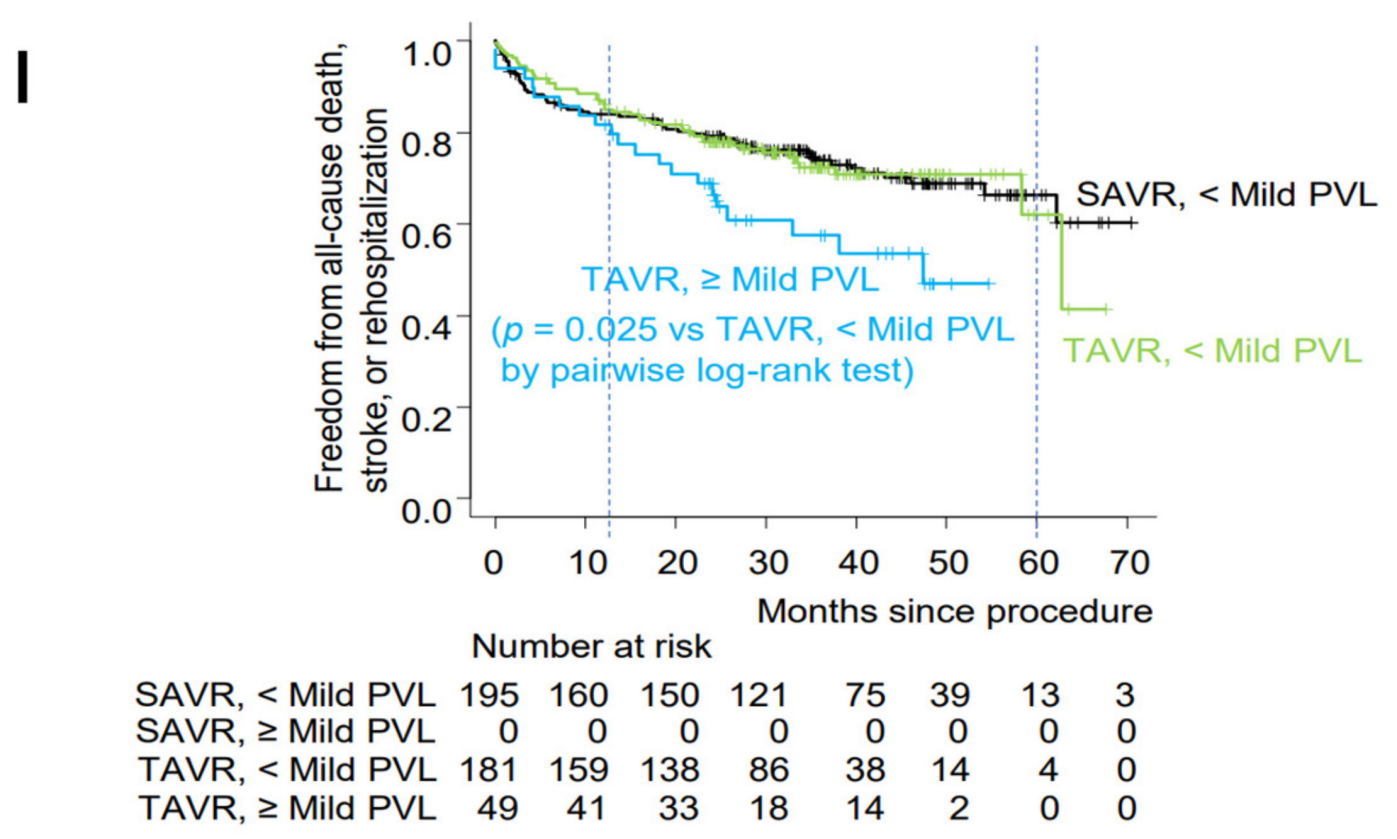
Kaplan–Meier curves showing freedom from all-cause death, stroke or rehospitalization. Patients were stratified by (**A**) study group, (**B**) with or without moderate or greater patient–prosthesis mismatch (PPM), (**C**) with or without moderate or greater PPM and study group, (**D**) with or without severe PPM, (**E**) with or without severe PPM and study group, (**F**) with or without paravalvular leakage (PVL), (**G**) with or without PVL and study group, (**H**) with or without mild or greater PVL, (**I**) with or without mild or greater PVL and study group. EOAI: effective orifice area index, NS: not significant, SAVR: surgical aortic valve replacement, TAVR: transcatheter aortic valve replacement.

There was no difference in freedom from all-cause death, stroke, or rehospitalization between the TAVR and SAVR groups (Figure 3A).

When the patients were stratified by EOAI, freedom from composite events was significantly higher in patients without PPM (EOAI ≥ 0.85 cm^2^/m^2^) than those with moderate or greater PPM (EOAI < 0.85 cm^2^/m^2^, *p* = 0.008, Figure 3B). In TAVR patients with moderate or greater PPM (TAVR and EOAI < 0.85 cm^2^/m^2^), freedom from composite events was even lower than that in SAVR patients with moderate or greater PPM (SAVR and EOAI < 0.85 cm^2^/m^2^), although without statistical significance (*p* = 0.154), and was significantly lower than that in TAVR patients without PPM (TAVR and EOAI ≥ 0.85 cm^2^/m^2^, *p* = 0.009, Figure 3C). In patients with severe PPM (EOAI < 0.65 cm^2^/m^2^), freedom from composite events was significantly lower than that in patients without severe PPM (EOAI ≥ 0.65 cm^2^/m^2^, *p* = 0.015, Figure 3D). Freedom from composite events was significantly lower in TAVR patients with severe PPM (TAVR and EOAI < 0.65 cm^2^/m^2^) than in TAVR patients without severe PPM (TAVR and EOAI ≥ 0.65 cm^2^/m^2^, *p* = 0.005, Figure 3E).

When the patients were stratified by the degree of PVL, there was no difference in freedom from composite events between patients with and without trivial or greater PVL (Figure 3F,G). However, in patients with mild or greater PVL, freedom from composite events was significantly lower than that in patients with PVL less than mild (*p* = 0.017, Figure 3H). Within TAVR group, patients with mild or greater PVL had significantly lower freedom from composite events than those with PVL less than mild (*p* = 0.025, Figure 3I).

### 3.5. Secondary Endpoints

Patient survival is shown in Supplementary Figure S1A–I. There was no difference in freedom from all-cause death between the TAVR and SAVR groups (Supplementary Figure S1A). Cumulative survival in patients with moderate or greater PPM (EOAI < 0.85 cm^2^/m^2^) tended to be lower than that in patients without PPM (EOAI ≥ 0.85 cm^2^/m^2^, *p* = 0.057). In TAVR patients with moderate or greater PPM (TAVR and EOAI < 0.85 cm^2^/m^2^), survival was significantly lower than that in TAVR patients without PPM (TAVR and EOAI ≥ 0.85 cm^2^/m^2^, *p* = 0.033, Supplementary Figure S1C). Patients with trivial or greater PVL had lower survival than those without PVL (*p* = 0.034, Supplementary Figure S1F).

Freedom from stroke is shown in Supplementary Figure S2A–I. There was no difference in freedom from stroke between the TAVR and SAVR groups (Supplementary Figure S2A). In TAVR patients with moderate or greater PPM (TAVR and EOAI ≥ 0.85 cm^2^/m^2^), freedom from stroke was significantly lower than that in TAVR patients without PPM (TAVR and EOAI < 0.85 cm^2^/m^2^, *p* = 0.025, Supplementary Figure S2C). Patients with severe PPM (EOAI < 0.65 cm^2^/m^2^) had a significantly lower freedom from stroke than those without severe PPM (EOAI ≥ 0.65 cm^2^/m^2^, *p* = 0.019, Supplementary Figure S2D). Freedom from stroke tended to be lower in patients with mild or greater PVL than in those with trivial or less PVL, although it did not reach statistical significance (Supplementary Figure S2H,I).

Freedom from rehospitalization is shown in Supplementary Figure S3A–I. There was no difference in the freedom from rehospitalization between the TAVR and SAVR groups (Supplementary Figure S3A). In patients with moderate or greater PPM (EOAI < 0.85 cm^2^/m^2^), freedom from rehospitalization tended to be lower than that in patients without PPM (EOAI ≥ 0.85 cm^2^/m^2^, *p* = 0.062, Supplementary Figure S3B). In TAVR patients with severe PPM (TAVR and EOAI < 0.65 cm^2^/m^2^), freedom from rehospitalization was significantly lower than that in SAVR patients with severe PPM (SAVR and EOAI < 0.65 cm^2^/m^2^, *p* = 0.032) and TAVR patients without severe PPM (TAVR and EOAI ≥ 0.65 cm^2^/m^2^, *p* < 0.001, Supplementary Figure S3E).

### 3.6. Risk Factors for Primary Endpoint

Among the preoperative factors, multivariable analysis using the Cox hazard model revealed that STS-PROM (hazard ratio 1.038, *p* = 0.042) and peripheral vascular disease (hazard ratio 1.595, *p* = 0.048) were significant risk factors for all-cause death, stroke, or rehospitalization (Table 4). Among the postoperative factors, permanent pacemaker implantation (hazard ratio 2.105, *p* = 0.022), newly induced renal replacement therapy (hazard ratio 2.811, *p* = 0.033), prosthetic valve endocarditis (hazard ratio 6.984, *p* < 0.001), duration of intensive care unit stay in days (hazard ratio 1.124, *p* = 0.006), and mild or greater PVL (hazard ratio 2.301, *p* = 0.019) were the significant risk factors for all-cause death, stroke, or rehospitalization (Table 5).

## 4. Discussions

This retrospective study focused on quantitative and qualitative echocardiographic outcomes after TAVR versus SAVR for severe AS. Postoperative echocardiographic study revealed superior valvular performance of TAVR to SAVR with higher EOAI and lower residual gradients. The lower rates of PPM after TAVR also support the higher effectiveness of treatment by TAVR. These findings are certainly counterbalanced by the significantly lower rates of paravalvular regurgitation and permanent pacemaker implantation in the SAVR group.

The superiority of TAVR to SAVR in valvular performance have already been demonstrated in previous reports, in which the rate of PPM was higher in SAVR than in TAVR, possibly due to the ability to oversize a transcatheter valve for a patient’s annular size [18,19,20]. In the present study, clinical outcome, shown as freedom from composite events of all-cause death, stroke, and rehospitalization, was significantly better in patients without PPM than in those with PPM (Figure 3B,D). These findings were also consistent with those of previous reports [13,14,15]. On the other hand, previous studies have also demonstrated that patients with a small aortic annulus who received TAVR still had a 4%–20% rate of severe PPM [18,19,20]. In this regard, PPM was observed in 8.7% of the TAVR patients in the present study. Oversized transcatheter valves in small aortic annulus patients with heavy asymmetric annular and left ventricular outflow tract calcification can increase the risk of annular rupture and coronary obstruction [21,22]. Furthermore, we have found that clinical outcomes of TAVR patients with PPM were not better (or even worse) than those of SAVR patients with PPM (Figure 3C,E). Therefore, SAVR may still play an important role in patients with small aortic annulus because surgical dilatation of the aortic annulus is possible, particularly in low-risk patients. Randomized studies are underway to compare TAVR with SAVR in patients with a small aortic annulus (VIVA Trial NCT 03383445).

The superiority of TAVR to SAVR was also demonstrated in its reduced invasiveness. In this regard, the amount of intraoperative bleeding and required transfusion was significantly lower in the TAVR group than in the SAVR group. New-onset atrial fibrillation was significantly more frequent in the SAVR group. The majority of TAVR patients (79.6%) were extubated in the operating room, compared with 0% of the SAVR patients. The duration of intensive care unit stay was also shorter in the TAVR group than in the SAVR group. These merits of TAVR have already been discussed in previous studies [23,24].

Despite these merits, PVL is one of the most serious drawbacks of the TAVR procedure. Recent data suggest that the presence of PVL, regardless of degree, can negatively affect clinical outcomes and neutralize the survival benefits of TAVI in patients with moderate or severe PVL [25]. PVL was associated with poor long-term procedural outcome, kidney injury, and life-threatening bleeding [26]. In the present study, patients with mild or more PVL had significantly worse clinical outcomes than those with less than mild PVL (Figure 3H). Additionally, mild or greater PVL was a significant risk factor for adverse events in multivariable Cox hazard model (Table 5). Newer generations of transcatheter valves are designed to reduce the occurrence of procedure-related complications, including PVL. They are generally designed to have better sealing with the native valve, with possibly no orifices left between the prosthesis and annulus, where PVL may occur [27].

Atrioventricular conduction disturbance, which requires permanent pacemaker implantation postoperatively, is another significant drawback of the TAVR procedure. In the present study, 23 patients (10.0%) in the TAVR group required permanent pacemaker implantation, which was significantly higher than that in the SAVR group (1.5%). Permanent pacemaker implantation was also detected as a risk factor for adverse events in the Cox hazard model (Table 5). Studies on how pacemaker implantation affects long-term outcomes in TAVR patients have provided mixed results, with some showing an increase in the 1-year mortality of up to 40% and a nearly doubled risk of heart failure admission [28,29,30,31]. While the risk of atrioventricular conduction disturbance alone should not dictate which procedure (TAVR or SAVR) should be indicated for severe AS patients, this potential risk should be discussed with patients, particularly in those who are young and have a low preoperative risk. These complications from TAVR may cause patients to undergo future procedures, such as generator changes and device upgrades for right ventricular pacing-induced cardiomyopathy. Younger and low-risk patients, who are at a higher risk of atrioventricular block, may elect to undergo SAVR to avoid these potential sequelae of TAVR [32].

### Study Limitations

The present study had several limitations, including its retrospective nature. As mentioned above, the indications for TAVR or SAVR were determined through discussion within the heart team and the preoperative characteristics of the patients were significantly different between the TAVR and SAVR groups. Therefore, it was impossible to compare the results of the present study and previously published industry-driven RCTs.

## 5. Conclusions

We have demonstrated that TAVR has superior valvular performance to SAVR in terms of postoperative EOAI. On the other hand, TAVR is associated with a more frequent and higher degree of PVL than SAVR. Although there was no difference in the freedom from all-cause death, stroke, or rehospitalization between TAVR and SAVR groups, the freedom from composite events was lower in patients with PPM or PVL than patients without these conditions. PVL and permanent pacemaker implantation were among significant risk factors for the composite events in the multivariable Cox hazard model. Newer-generation prostheses in TAVR and additional procedures in SAVR, such as annulus enlargement, are expected to improve the clinical outcomes in both groups. Future research should focus on the impact of these new devices and additional procedures on clinical outcomes.

## Figures and Tables

**Table 1:** Preoperative characteristics of the patients.

	TAVR	SAVR	
Characteristics	(*n* = 230)	(*n* = 195)	*p*-Value
Age, year	84.6 ± 4.3	74.0 ± 8.0	<0.001
Female sex, no. (%)	166 (72.2)	82 (42.1)	<0.001
Body mass index	22.2 ± 3.7	23.4 ± 3.9	<0.001
STS-PROM	6.6 ± 4.6	5.1 ± 5.5	<0.001
NYHA class III or IV, no. (%)	60 (26.1)	55 (28.2)	0.662
Coronary artery disease, no. (%)	33 (14.3)	69 (32.3)	<0.001
Triple vessel disease and/or left main trunk disease, no (%)	3 (1.3)	23 (11.8)	<0.001
Cerebral vascular disease/Carotid disease, no. (%)	60 (26.1)	32 (16.4)	0.018
Peripheral vascular disease, no. (%)	31 (13.5)	38 (19.5)	0.113
COPD, no. (%)	42 (18.3)	33 (16.9)	0.799
Creatinine > 2 mg/dL, no. (%)	2 (0.9)	39 (20.0)	<0.001
Hemodialysis, no. (%)	0 (0)	30 (15.4)	<0.001
Diabetes, no. (%)	76 (33.0)	70 (35.9)	0.541
Atrial fibrillation, no (%)	33 (14.3)	33 (16.9)	0.503
Previous cardiovascular surgery, no. (%)	12 (5.2)	9 (4.6)	0.826
Bicuspid aortic valve, no. (%)	6 (2.6)	39 (20.0)	<0.001
Left ventricular ejection fraction, %	58.1 ± 11.4	55.7 ± 13.1	0.043
Left ventricular ejection fraction < 30%, no. (%)	3 (1.3)	11 (5.6)	0.014
Emergent/Urgent operation, no. (%)	8 (3.5)	23 (11.8)	0.001
Concomitant CABG/TAVR + PCI, no. (%)	27 (11.7)	57 (29.2)	<0.001

AVR: transcatheter aortic valve replacement, CABG: coronary artery bypass grafting, COPD: chronic occlusive pulmonary disease, NYHA: New York Heart Association, PCI: percutaneous coronary intervention, SAVR: surgical aortic valve replacement, STS-PROM: Society of Thoracic Surgery-Predicted Risk of Mortality.

**Table 2:** Prosthesis used in this study.

	Size (mm)								
Prosthesis	19	20	21	22	23	25	26	27	29
**Transcatheter aortic valve replacement**									
Sapien XT	-	1	-	-	22	-	11	-	1
Sapien 3	-	5	-	-	92	-	38	-	11
CoreValve	-	0	-	-	0	-	1	-	1
CoreValve EVOLUTE R	-	0	-	-	4	-	18	-	13
CoreValve EVOLUTE PRO	-	0	-	-	1	-	6	-	5
**Surgical aortic valve replacement Biological**									
Magna Ease	15	-	28	-	13	8	-	0	0
INSPIRIS RESILIA	3	-	2	-	7	0	-	0	0
Carpentier-Edwards PERIMOUNT	0	-	1	-	2	1	-	1	0
Crown PRT	4	-	32	-	39	6	-	0	0
SOLO SMART	1	-	4	-	3	0	-	0	0
Mitroflow	2	-	0	-	0	0	-	0	0
Trifecta	1	-	3	-	0	0	-	0	0
Mosaic Ultra	0	-	0	-	2	0	-	0	0
AVALUS	1	-	0	-	3	1	-	1	0
**Mechanical**									
SJM Regent	3	-	2	-	1	0	-	0	0
ATS	-	2	-	1	-	-	0	-	-
On-X	0	-	0	-	2	0	-	0	0

**Table 3:** Clinical outcomes.

	TAVR	SAVR	
Parameters	(*n* = 230)	(*n* = 195)	*p*-Value
Intra-aortic balloon pump, no. (%)	3 (1.3)	14 (7.2)	0.002
Extracorporeal membrane oxygenation, no. (%)	1 (0.4)	0 (0.0)	1
Intraoperative bleeding, mL	116.0 ± 342.6	861.0 ± 615.1	<0.001
Transfusion (red blood cell), mL	338.1 ± 349.4	1063.4 ± 673.9	<0.001
Reoperation for bleeding, no. (%)	7 (3.0)	11 (5.6)	0.229
New-onset atrial fibrillation, no. (%)	13 (5.7)	59 (30.3)	<0.001
Permanent pacemaker implantation, no. (%)	23 (10.0)	3 (1.5)	<0.001
Newly induced renal replacement therapy, no. (%)	3 (1.3)	5 (2.6)	0.479
Prosthetic valve endocarditis, no. (%)	1 (0.4)	6 (3.1)	0.051
Peripheral vascular complication, no. (%)	15 (6.5)	0 (0.0)	<0.001
Extubation in operation room, no. (%)	183 (79.6)	0 (0.0)	<0.001
Intubation time, hours	9.1 ± 51.3	17.4 ± 29.3	0.049
Intensive care unit stay, days	1.4 ± 3.5	3.2 ± 3.8	<0.001
Echocardiographic findings			
Peak velocity through aortic valve, m/s	2.1 ± 0.5	2.3 ± 0.5	<0.001
Mean pressure gradient, mmHg	10.0 ± 4.8	11.6 ± 4.9	<0.001
Peak pressure gradient, mmHg	18.5 ± 8.5	21.4 ± 8.7	<0.001
Effective orifice area index, cm^2^/m^2^	1.27 ± 0.35	1.06 ± 0.27	<0.001
Effective orifice area index < 0.85 cm^2^/m^2^, no. (%)	20 (8.7)	44 (22.6)	<0.001
Effective orifice area index < 0.65 cm^2^/m^2^, no. (%)	2 (0.9)	7 (3.6)	0.087
≥Trivial paravalvular leakage, no. (%)	201 (87.4)	16 (8.2)	<0.001
≥Mild paravalvular leakage, no. (%)	49 (21.3)	0 (0.0)	<0.001

SAVR: surgical aortic valve replacement, TAVR: transcatheter aortic valve replacement.

**Table 4:** Preoperative risk factors for the primary endpoint (Cox hazard model).

	Univariate	Multivariable	
Parameters	*p*-Value	*p*-Value	Hazard Ratio (95% CI)
Age	0.763		
Female sex	0.135		
Body-mass index	0.671		
STS-PROM	<0.001	0.042	1.038 (1.001–1.076)
NYHA class III or IV	0.046	0.721	0.921 (0.587–1.445)
Coronary artery disease	0.036	0.7	1.130 (0.607–2.105)
Triple vessel disease and/or left main trunk disease	0.449		
Cerebral vascular disease/Carotid disease	0.7		
Peripheral vascular disease	<0.001	0.048	1.595 (1.004–2.534)
COPD	0.802		
Creatinine > 2 mg/dL	<0.001	0.707	1.190 (0.481–2.946)
Hemodialysis	<0.001	0.487	1.398 (0.544–3.595)
Diabetes	0.186		
Atrial fibrillation	0.032	0.066	1.524 (0.973–2.388)
Previous cardiovascular surgery	0.531		
Bicuspid aortic valve	0.832		
Left ventricular ejection fraction	0.263		
Left ventricular ejection fraction < 30%	0.092	0.432	1.424 (0.590–3.433)
Emergent/Urgent operation	0.284		
Concomitant CABG/TAVR+PCI	0.082	0.844	1.069 (0.553–2.065)
TAVR	0.36		

CABG: coronary artery bypass grafting, COPD: chronic occlusive pulmonary disease, NYHA: New York Heart As-sociation, PCI: percutaneous coronary intervention, STS-PROM: Society of Thoracic Surgery-Predicted Risk of Mortality, TAVR: transcatheter aortic valve replacement.

**Table 5:** Postoperative risk factors for the primary endpoint (Cox hazard model).

	Univariate	Multivariable	
Parameters	*p*-Value	*p*-Value	Hazard Ratio (95% CI)
Intra-aortic balloon pump	0.056	0.438	1.389 (0.606–3.183)
Extracorporeal membrane oxygenation	0.994		
Intraoperative bleeding (L)	0.026	0.926	1.016 (0.723–1.429)
Transfusion (red blood cell) (L)	0.095	0.469	1.143 (0.796–1.641)
Reoperation for bleeding	0.72		
Newly onset atrial fibrillation	0.303		
Permanent pacemaker implantation	0.022	0.032	2.105 (1.066–4.154)
Newly induced renal replacement therapy	<0.001	0.033	2.811 (1.085–7.280)
Prosthetic valve endocarditis	<0.001	<0.001	6.984 (3.094–15.770)
Peripheral vascular complication	0.204		
Intubation time (h)	0.071	0.072	0.995 (0.989–1.000)
Intensive care unit stay (days)	<0.001	0.006	1.124 (1.035–1.221)
Echocardiographic findings			
Peak velocity through aortic valve (m/s)	0.51		
Mean pressure gradient (mmHg)	0.87		
Peak pressure gradient (mmHg)	0.821		
Effective orifice area index (cm^2^/m^2^)	0.701		
Effective orifice area index < 0.85 cm^2^/m^2^	0.009	0.371	1.269 (0.753–2.140)
Effective orifice area index < 0.65 cm^2^/m^2^	0.02	0.17	2.072 (0.731–5.873)
≥Trivial paravalvular leakage	0.2		
≥Mild paravalvular leakage	0.019	0.001	2.301 (1.381–3.834)

## Data Availability

This study is not a clinical trial.

## Supplementary Materials

The following supporting information can be downloaded at: https://www.mdpi.com/article/10.3390/jcdd9100355/s1, Figure S1: patient survival; Figure S2: freedom from stroke; Figure S3: freedom from rehospitalization.
