# Supplementary material for: Prosthetic Valve Function after Aortic Valve Replacement for Severe Aortic Stenosis by Transcatheter Procedure versus Surgery
Source: J Cardiovasc Dev Dis. 2022 Oct 16;9(10):355. doi: 10.3390/jcdd9100355 (PMC9604414; doi:10.3390/jcdd9100355)
Supplement: Supplementary file 1 [file jcdd-09-00355-s001.zip › jcdd-1946443-supplementary .pdf]

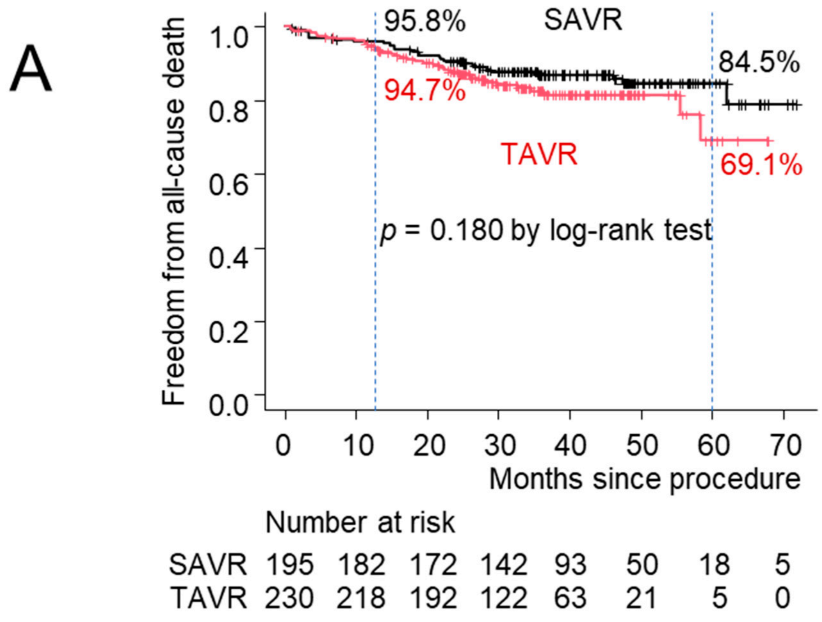
A
95.8%
SAVR
84.5%
94.7%
TAVR
69.1%
p = 0.180 by log-rank test
Freedom from all-cause death
1.0
0.8
0.6
0.4
0.2
0.0
0
10
20
30
40
50
60
70
Months since procedure
Number at risk
SAVR 195 182 172 142 93 50 18 5
TAVR 230 218 192 122 63 21 5 0

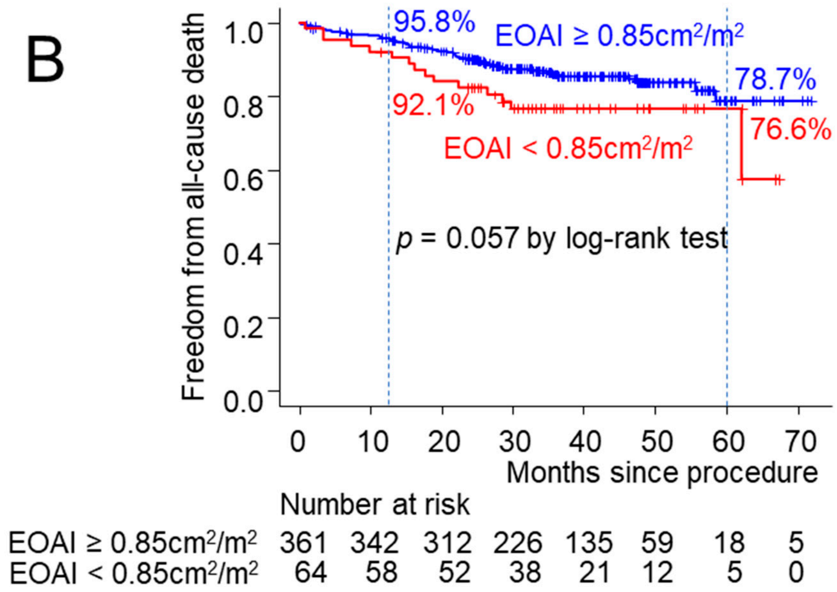
B
95.8%
EOAI ≥ 0.85cm2/m2
78.7%
92.1%
76.6%
EOAI < 0.85cm2/m2
p = 0.057 by log-rank test
Freedom from all-cause death
1.0
0.8
0.6
0.4
0.2
0.0
0
10
20
30
40
50
60
70
Months since procedure
Number at risk
EOAI ≥ 0.85cm2/m2 361 342 312 226 135 59 18 5
EOAI < 0.85cm2/m2 64 58 52 38 21 12 5 0

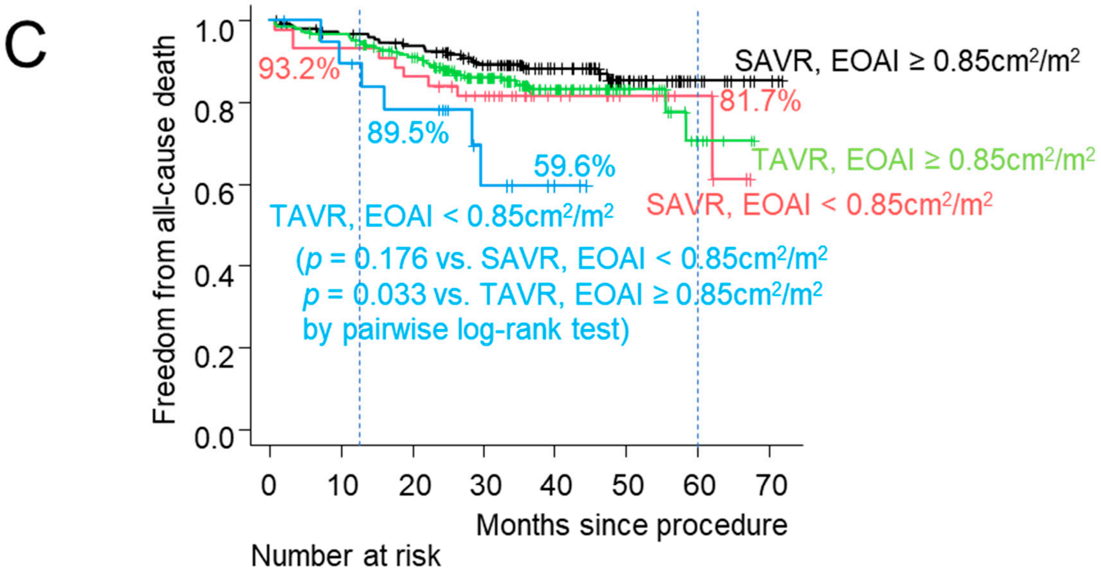

Number at risk

SAVR, EOAI ≥ 0.85cm^2/m^2	151	141	134	110	74	38	13	5
SAVR, EOAI < 0.85cm^2/m^2	44	41	38	32	19	12	5	0
TAVR, EOAI ≥ 0.85cm^2/m^2	210	201	178	116	61	21	5	0
TAVR, EOAI < 0.85cm^2/m^2	20	17	14	6	2	0	0	0

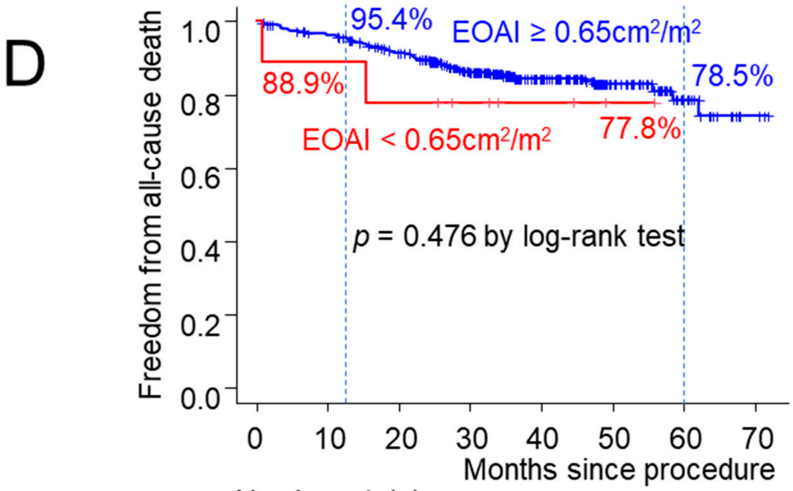

Number at risk

EOAI ≥ 0.65cm^2/m^2	416	392	357	259	153	70	23	5
EOAI < 0.65cm^2/m^2	9	8	7	5	3	1	0	0

E

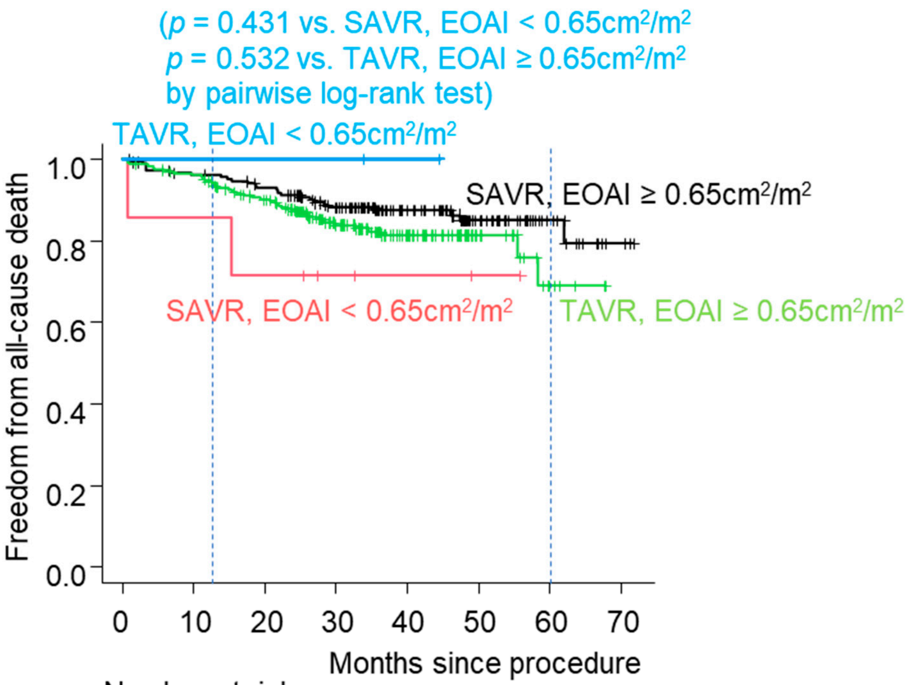

Number at risk

SAVR, EOAI ≥ 0.65cm²/m²	188	176	167	139	91	49	13	5
SAVR, EOAI < 0.65cm²/m²	7	6	5	3	2	1	0	0
TAVR, EOAI ≥ 0.65cm²/m²	228	216	190	120	62	21	5	0
TAVR, EOAI < 0.65cm²/m²	2	2	2	2	1	0	0	0

F

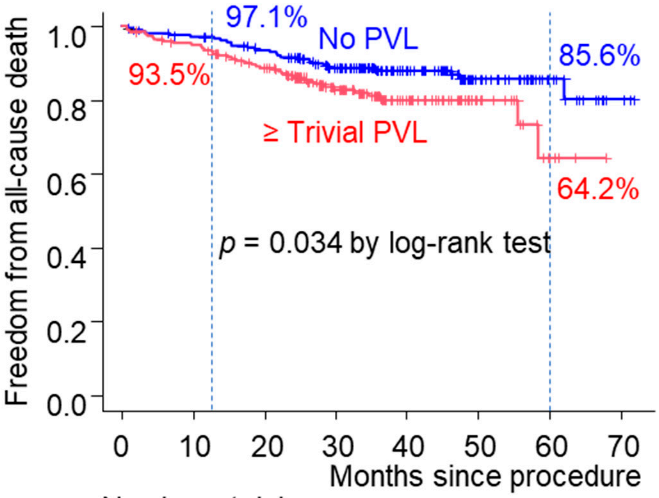

Number at risk

No PVL	208	197	184	150	96	50	19	5
≥ Trivial PVL	217	203	180	114	60	21	4	0

G

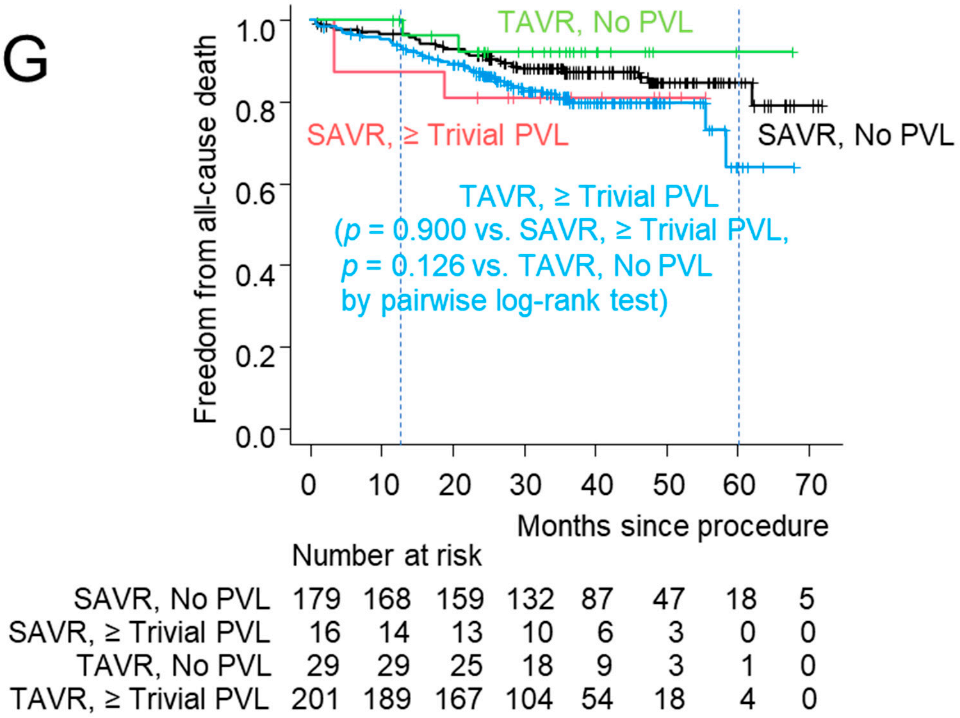
TAVR, No PVL
SAVR, ≥ Trivial PVL
SAVR, No PVL
TAVR, ≥ Trivial PVL
(p = 0.900 vs. SAVR, ≥ Trivial PVL,
p = 0.126 vs. TAVR, No PVL
by pairwise log-rank test)
Freedom from all-cause death
Months since procedure
Number at risk
SAVR, No PVL 179 168 159 132 87 47 18 5
SAVR, ≥ Trivial PVL 16 14 13 10 6 3 0 0
TAVR, No PVL 29 29 25 18 9 3 1 0
TAVR, ≥ Trivial PVL 201 189 167 104 54 18 4 0

H

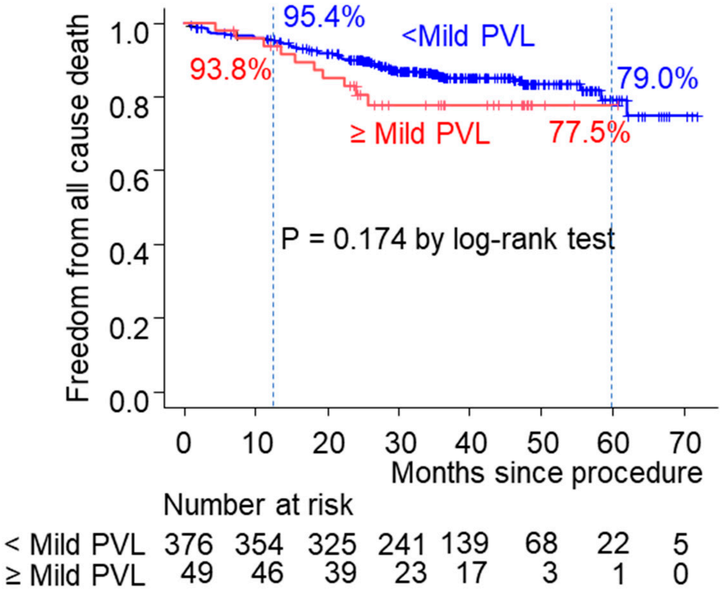
95.4%
<Mild PVL
93.8%
79.0%
≥ Mild PVL
77.5%
P = 0.174 by log-rank test
Freedom from all cause death
Months since procedure
Number at risk
< Mild PVL 376 354 325 241 139 68 22 5
≥ Mild PVL 49 46 39 23 17 3 1 0

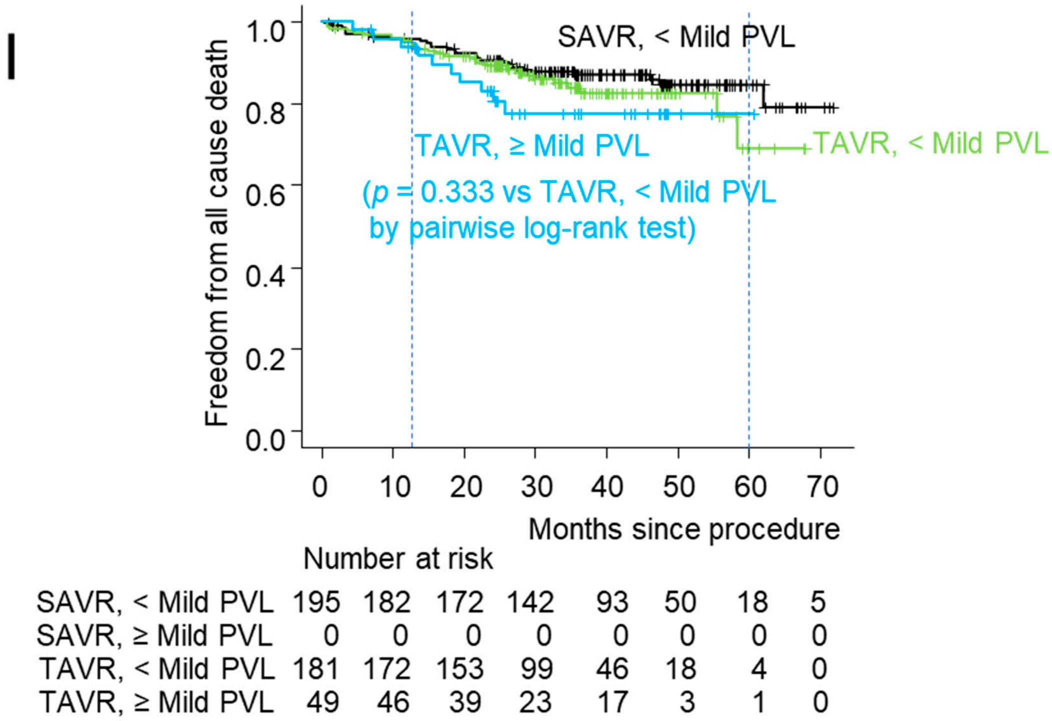

Figure S1. patient survival.

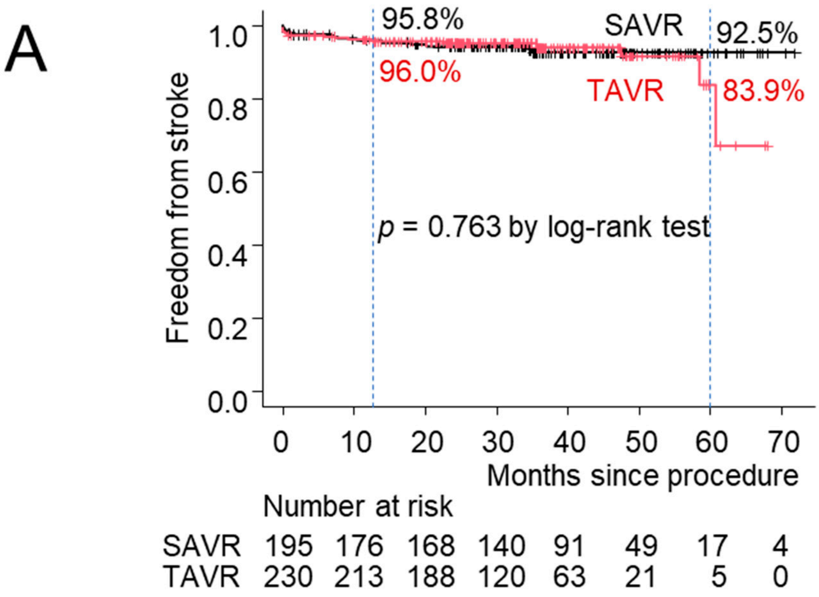

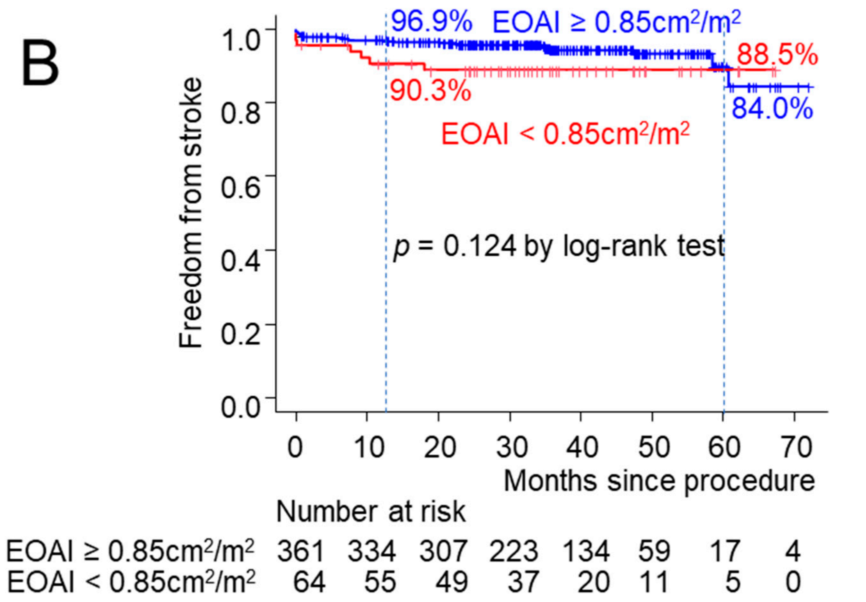
B
Freedom from stroke
1.0
0.8
0.6
0.4
0.2
0.0
96.9% EOAI ≥ 0.85cm²/m²
88.5%
90.3%
84.0%
EOAI < 0.85cm²/m²
p = 0.124 by log-rank test
0 10 20 30 40 50 60 70
Months since procedure
Number at risk
EOAI ≥ 0.85cm²/m² 361 334 307 223 134 59 17 4
EOAI < 0.85cm²/m² 64 55 49 37 20 11 5 0

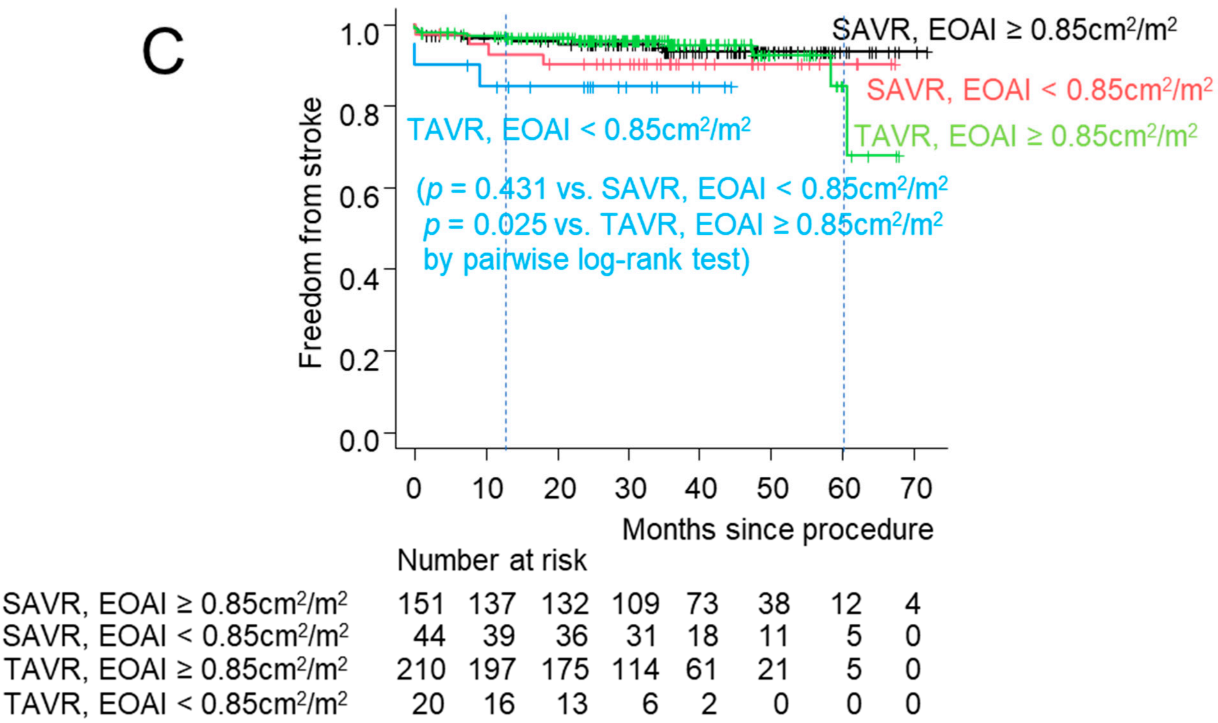
C
Freedom from stroke
1.0
0.8
0.6
0.4
0.2
0.0
SAVR, EOAI ≥ 0.85cm²/m²
SAVR, EOAI < 0.85cm²/m²
TAVR, EOAI ≥ 0.85cm²/m²
TAVR, EOAI < 0.85cm²/m²
(p = 0.431 vs. SAVR, EOAI < 0.85cm²/m²
p = 0.025 vs. TAVR, EOAI ≥ 0.85cm²/m²
by pairwise log-rank test)
0 10 20 30 40 50 60 70
Months since procedure
Number at risk
SAVR, EOAI ≥ 0.85cm²/m² 151 137 132 109 73 38 12 4
SAVR, EOAI < 0.85cm²/m² 44 39 36 31 18 11 5 0
TAVR, EOAI ≥ 0.85cm²/m² 210 197 175 114 61 21 5 0
TAVR, EOAI < 0.85cm²/m² 20 16 13 6 2 0 0 0

D

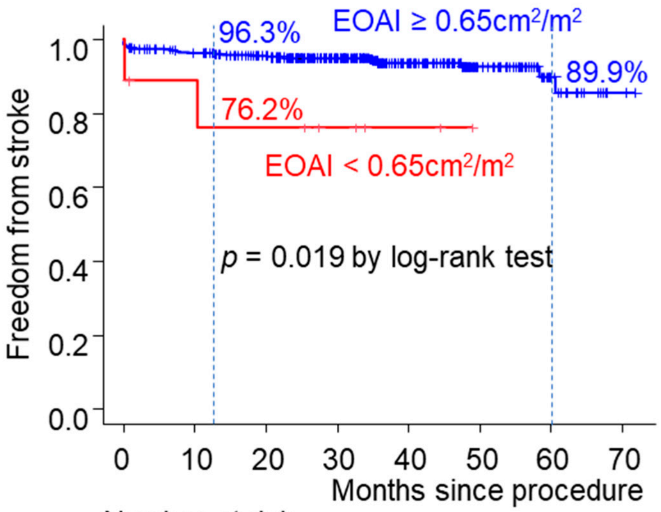

Number at risk

EOAI ≥ 0.65cm²/m²	416	382	350	256	152	70	22	4
EOAI < 0.65cm²/m²	9	7	6	4	2	0	0	0

E

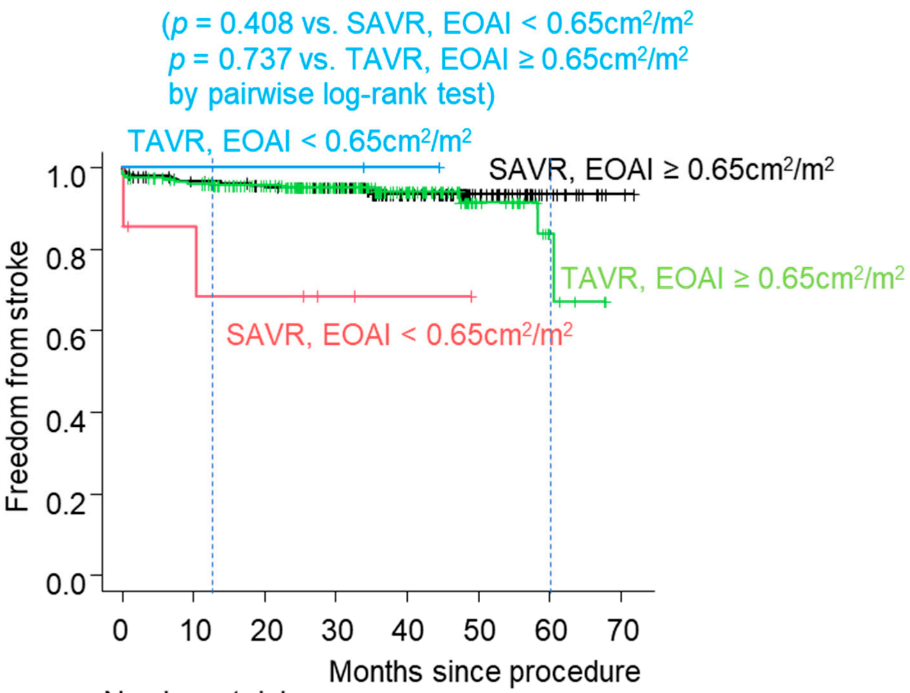

Number at risk

SAVR, EOAI ≥ 0.65cm²/m²	158	171	164	138	90	49	17	4
SAVR, EOAI < 0.65cm²/m²	7	5	4	2	1	0	0	0
TAVR, EOAI ≥ 0.65cm²/m²	228	211	186	118	62	21	5	0
TAVR, EOAI < 0.65cm²/m²	2	2	2	2	1	0	0	0

F

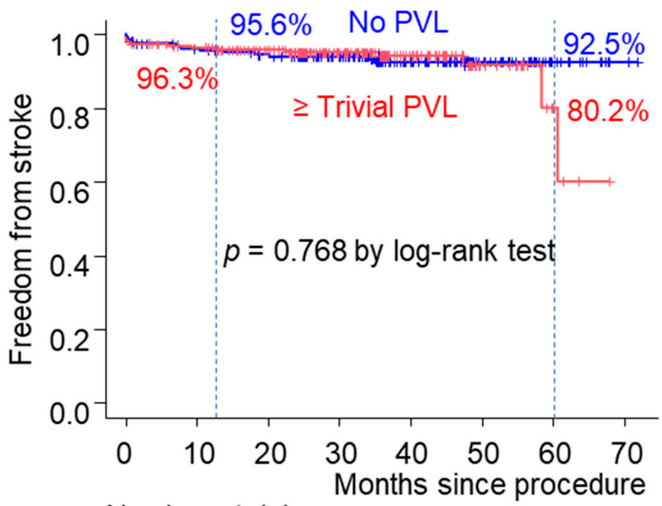

Number at risk

	0	10	20	30	40	50	60	70
No PVL	208	190	180	148	94	50	18	4
≥ Trivial PVL	217	199	176	112	60	20	4	0

G

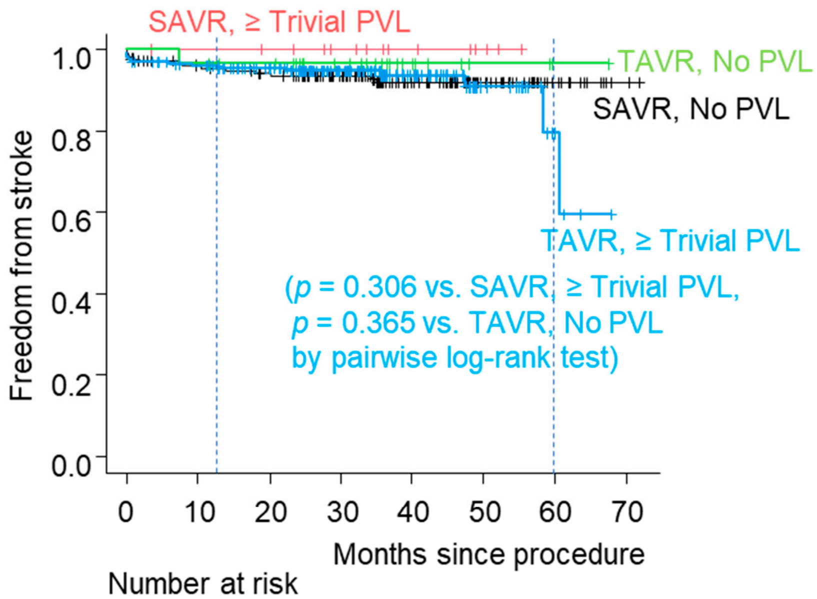

Number at risk

	0	10	20	30	40	50	60	70
SAVR, No PVL	179	162	155	130	85	46	17	4
SAVR, ≥ Trivial PVL	16	14	13	10	6	3	0	0
TAVR, No PVL	29	28	25	18	9	4	1	0
TAVR, ≥ Trivial PVL	201	185	163	102	54	17	4	0

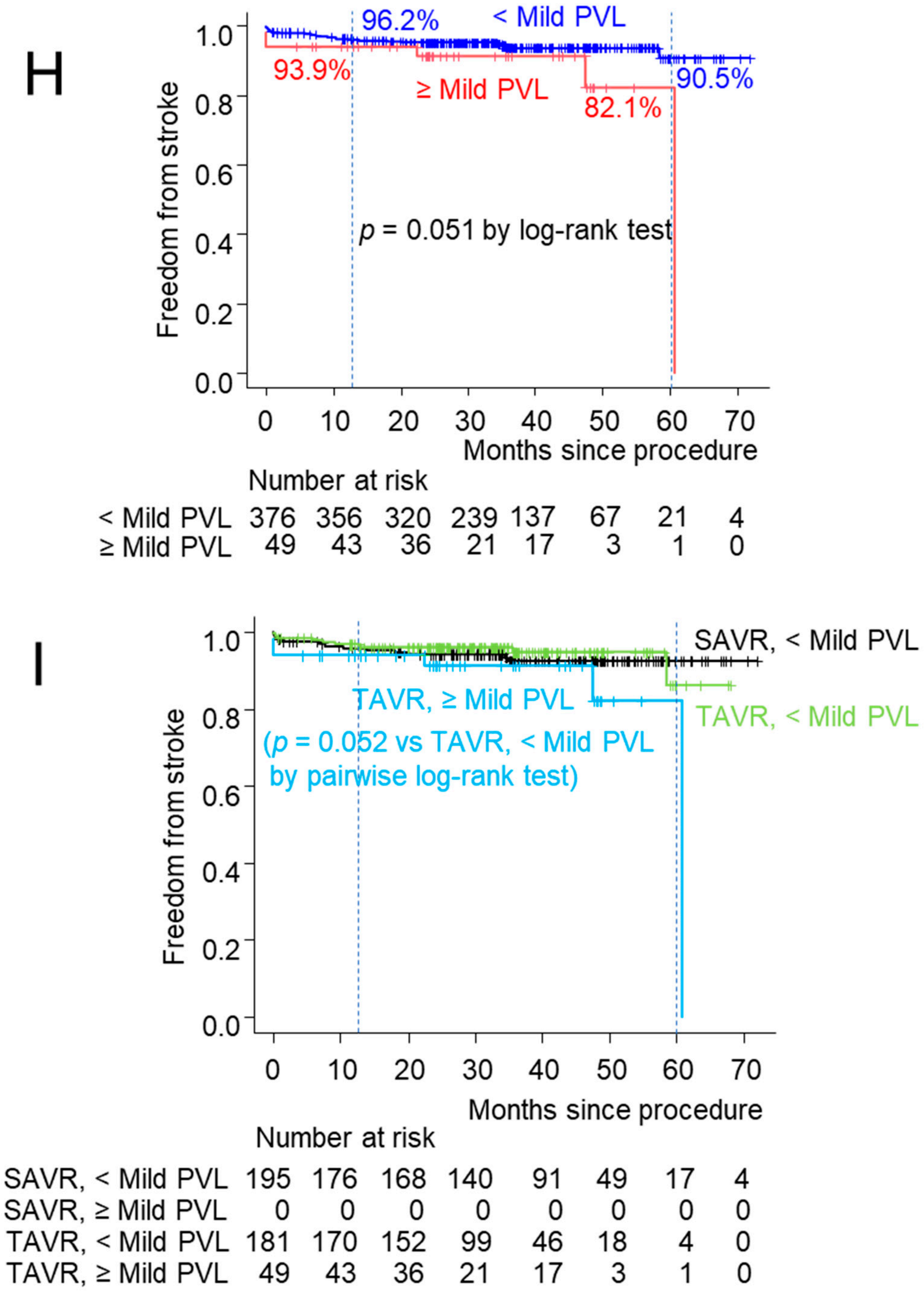

Figure S2. freedom from stroke.

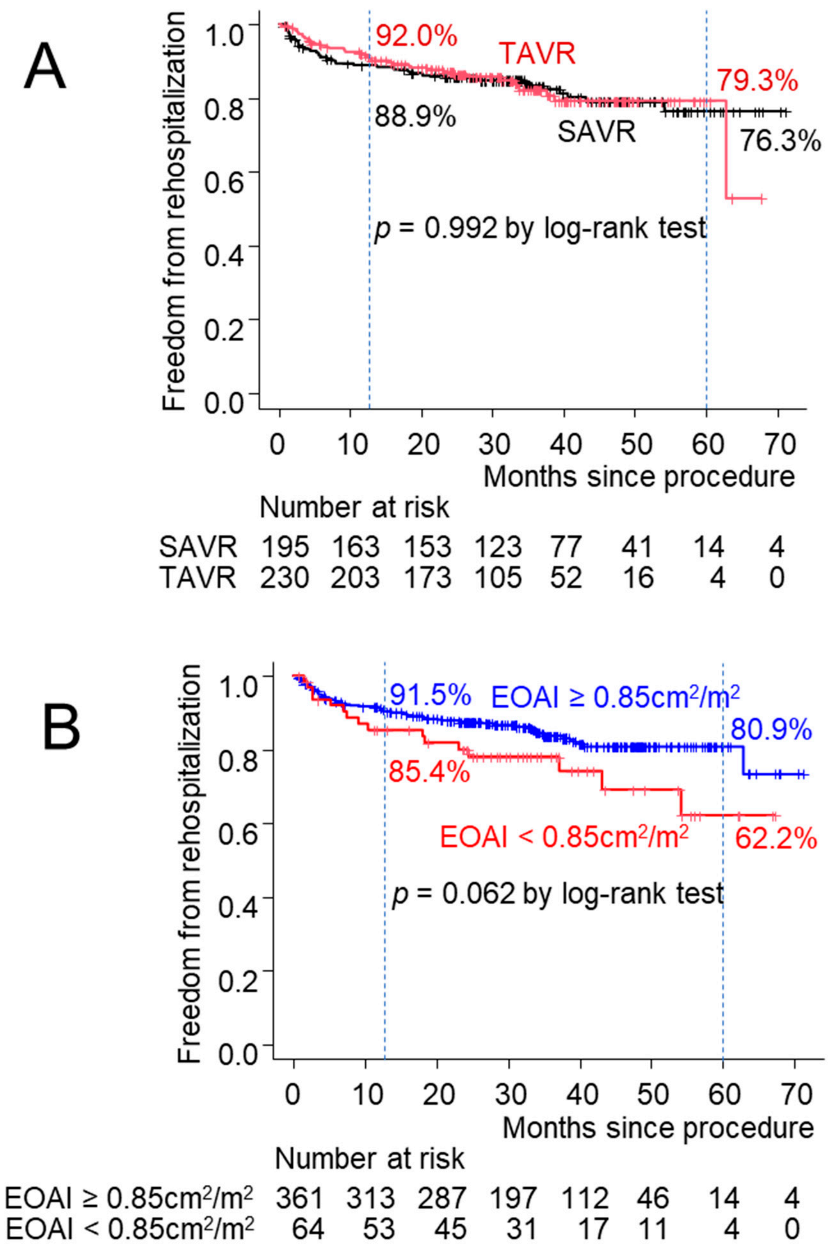
A
Freedom from rehospitalization
1.0
0.8
0.6
0.4
0.2
0.0
92.0%
TAVR
79.3%
88.9%
SAVR
76.3%
p = 0.992 by log-rank test
0
10
20
30
40
50
60
70
Months since procedure
Number at risk
SAVR 195 163 153 123 77 41 14 4
TAVR 230 203 173 105 52 16 4 0
B
Freedom from rehospitalization
1.0
0.8
0.6
0.4
0.2
0.0
91.5%
EOAI ≥ 0.85cm2/m2
80.9%
85.4%
EOAI < 0.85cm2/m2
62.2%
p = 0.062 by log-rank test
0
10
20
30
40
50
60
70
Months since procedure
Number at risk
EOAI ≥ 0.85cm2/m2 361 313 287 197 112 46 14 4
EOAI < 0.85cm2/m2 64 53 45 31 17 11 4 0

C

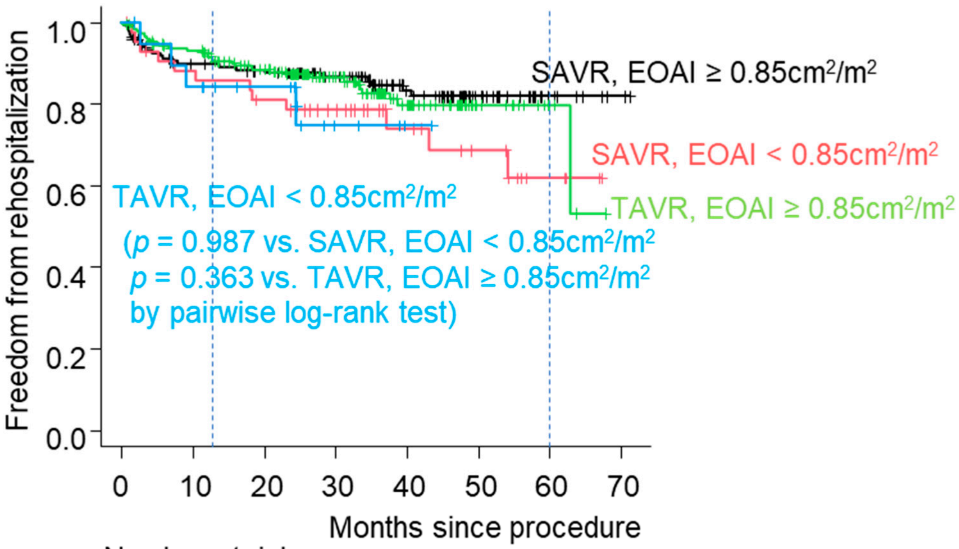

Number at risk

SAVR, EOAI ≥ $0.85 cm^2/m^2$	151	126	120	96	61	30	10	4
SAVR, EOAI < $0.85 cm^2/m^2$	44	37	33	27	16	11	4	0
TAVR, EOAI ≥ $0.85 cm^2/m^2$	210	187	161	101	51	16	4	0
TAVR, EOAI < $0.85 cm^2/m^2$	20	16	12	4	1	0	0	0

D

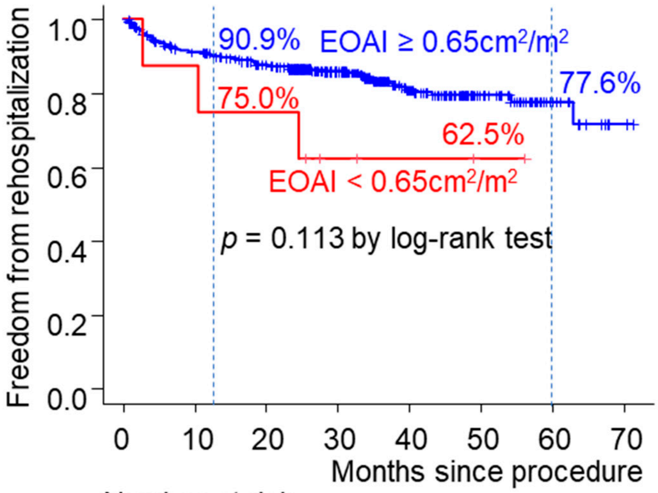

Number at risk

EOAI ≥ $0.65 cm^2/m^2$	416	359	320	225	127	56	18	4
EOAI < $0.65 cm^2/m^2$	9	7	6	3	2	1	0	0

E

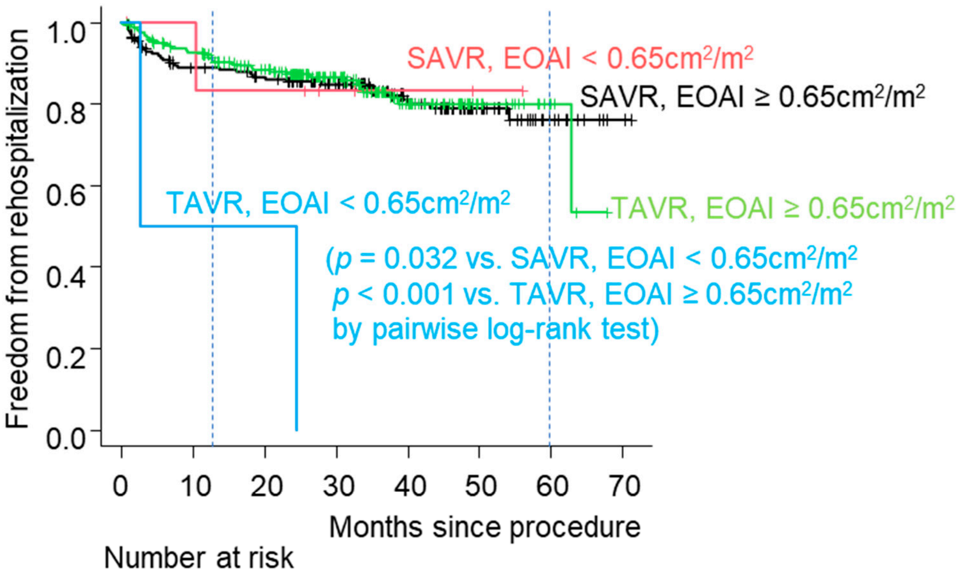

Number at risk

SAVR, EOAI ≥ 0.65cm²/m²	188	157	148	120	75	40	14	4
SAVR, EOAI < 0.65cm²/m²	7	6	5	3	2	1	0	0
TAVR, EOAI ≥ 0.65cm²/m²	228	202	172	105	52	16	4	0
TAVR, EOAI < 0.65cm²/m²	2	1	1	0	0	0	0	0

F

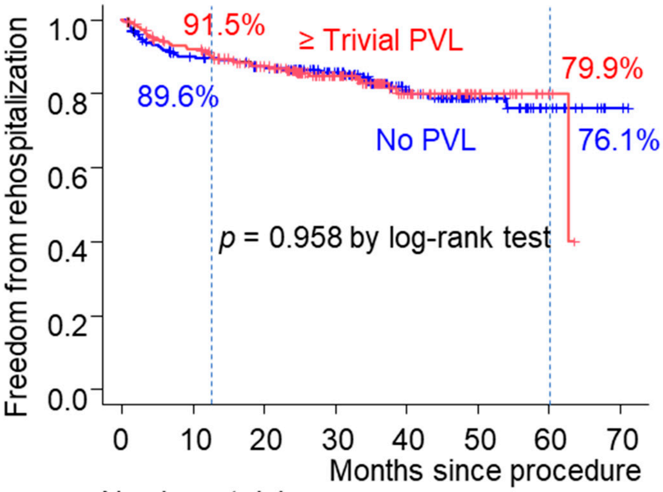

Number at risk

No PVL	208	176	164	131	79	40	15	4
≥ Trivial PVL	217	190	162	97	50	17	3	0

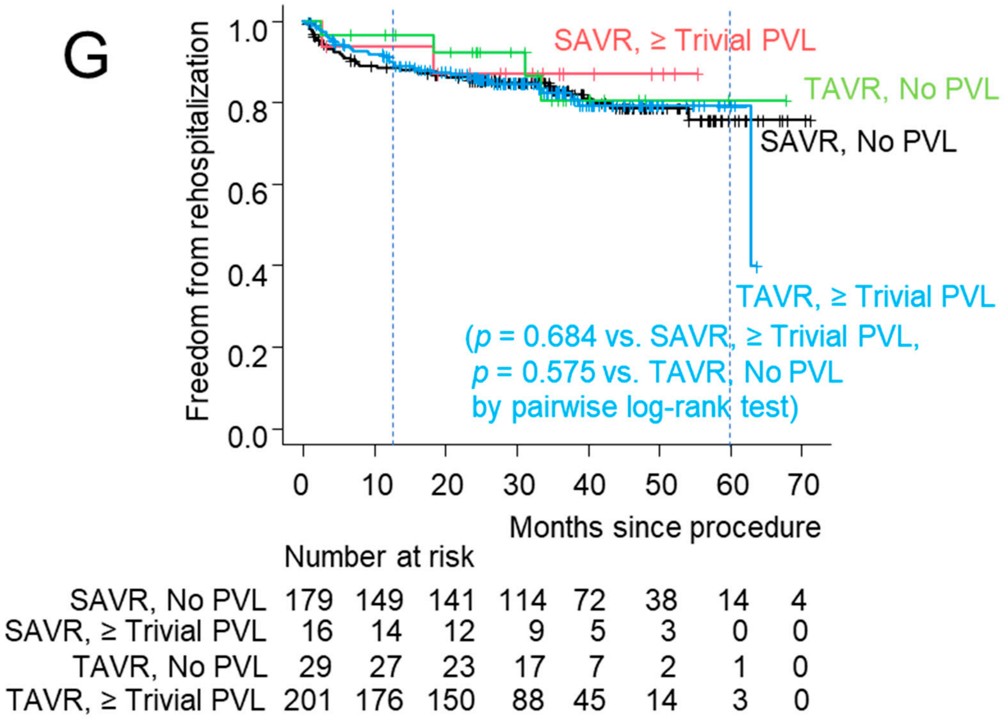
G
Freedom from rehospitalization
1.0
0.8
0.6
0.4
0.2
0.0
SAVR, ≥ Trivial PVL
TAVR, No PVL
SAVR, No PVL
TAVR, ≥ Trivial PVL
(p = 0.684 vs. SAVR, ≥ Trivial PVL,
p = 0.575 vs. TAVR, No PVL
by pairwise log-rank test)
0 10 20 30 40 50 60 70
Months since procedure
Number at risk
SAVR, No PVL 179 149 141 114 72 38 14 4
SAVR, ≥ Trivial PVL 16 14 12 9 5 3 0 0
TAVR, No PVL 29 27 23 17 7 2 1 0
TAVR, ≥ Trivial PVL 201 176 150 88 45 14 3 0

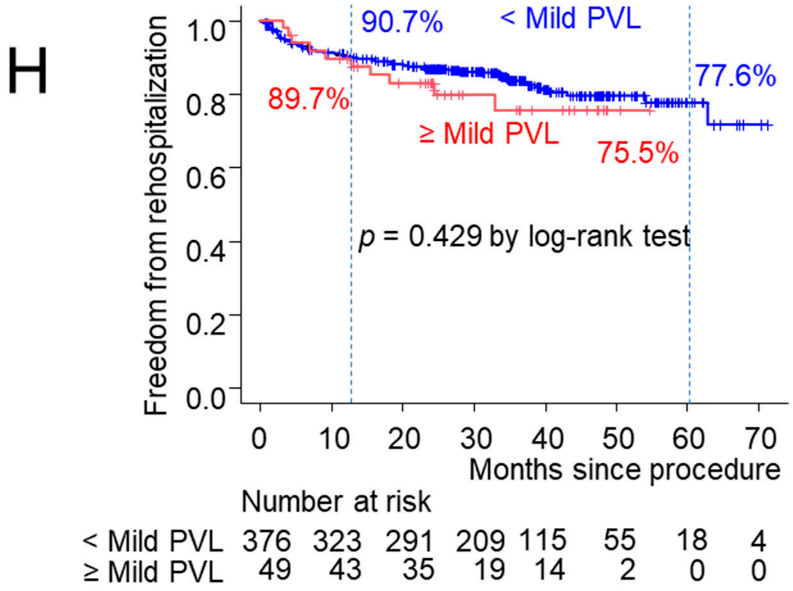
H
Freedom from rehospitalization
1.0
0.8
0.6
0.4
0.2
0.0
90.7%
< Mild PVL
77.6%
89.7%
≥ Mild PVL
75.5%
p = 0.429 by log-rank test
0 10 20 30 40 50 60 70
Months since procedure
Number at risk
< Mild PVL 376 323 291 209 115 55 18 4
≥ Mild PVL 49 43 35 19 14 2 0 0

I

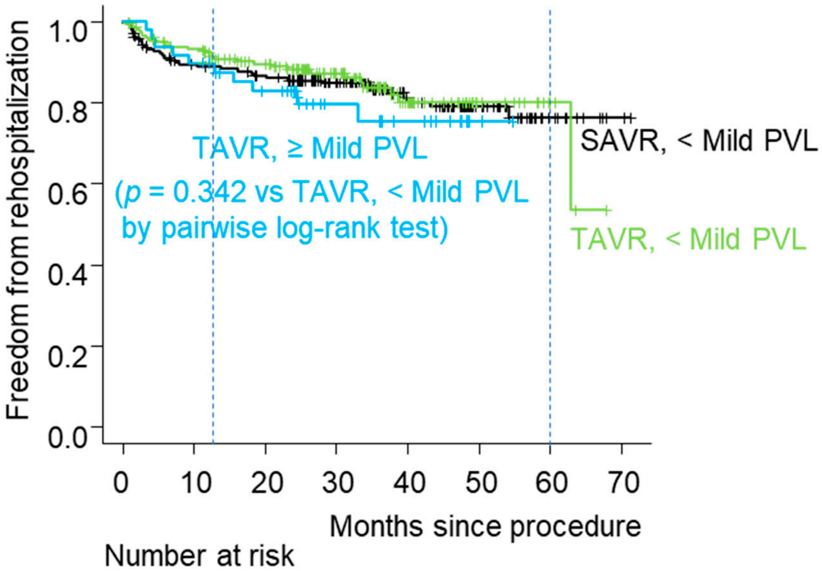

Number at risk

SAVR, < Mild PVL	195	163	153	123	77	41	14	4
SAVR, ≥ Mild PVL	0	0	0	0	0	0	0	0
TAVR, < Mild PVL	181	160	138	86	38	14	4	0
TAVR, ≥ Mild PVL	49	43	35	19	14	2	0	0

Figure S3. freedom from rehospitalization.
